# Sodium glucose transporter‐2 inhibition has no renoprotective effects on non‐diabetic chronic kidney disease

**DOI:** 10.14814/phy2.13228

**Published:** 2017-03-31

**Authors:** Qiuyue Ma, Stefanie Steiger, Hans‐Joachim Anders

**Affiliations:** ^1^ Medizinische Klinik und Poliklinik IVKlinikum der Universität MünchenMunichGermany

**Keywords:** Crystal, diabetic nephropathy, glomerulosclerosis, progression

## Abstract

Sodium glucose transporter (SGLT)‐2 inhibition has renoprotective effects in diabetic kidney disease. Whether similar effects can be achieved also in non‐diabetic kidney disease is speculative. Chronic kidney disease was induced in C57BL/6N mice by feeding an oxalate‐rich diet for 14 days, known to induce nephrocalcinosis‐related tubular atrophy and interstitial fibrosis without directly affecting the glomerular compartment. Empagliflozin treatment started from day 0 of oxalate feeding had no effect on the decline of glomerular filtration rate, crystal deposition, blood urea nitrogen or serum creatinine levels on day 7 and 14. Tissue morphometry of tubular injury and kidney mRNA levels of kidney injury molecule‐1 or tissue inhibitor of metalloproteinase‐2 were comparable between empagliflozin‐ and vehicle‐treated mice with oxalate nephropathy on day 7 and 14. Similarly, empagliflozin did not affect markers of interstitial fibrosis, including silver, alpha smooth muscle actin (*α* SMA) and collagen 1 staining, and mRNA levels of fibronectin‐1, collagen 1*α*1, fibroblast‐specific protein‐1, and transforming growth factor (TGF)‐*β*2 on day 7 and 14. Thus, the specific renoprotective mechanisms‐of‐action of SGLT2 inhibition in diabetic kidney disease do not apply to chronic oxalosis, a non‐diabetic form of chronic kidney disease.

## Introduction

Chronic kidney disease (CKD) is a global health care problem of increasing dimension (Eckardt et al. 2013). CKD progression is the result of progressive loss of nephrons being replaced by fibrous extracellular matrix components, that is interstitial fibrosis, which stabilizes the remaining nephrons. Therapeutic interventions aiming to retard or stop the progressive loss of nephrons and the associated decline of renal excretory function can be divided into two categories: Firstly, abolishing the specific cause of disease, an approach that is feasible in renal infections or toxic kidney injury, which is still impossible in genetic kidney disease or aging nephropathy, and which is notoriously difficult in complex disease states, such as metabolic syndrome. Secondly, targeting unspecific mechanisms of CKD progression, for example reducing glomerular hyperfiltration and hypertension with inhibitors of the renin‐angiotensin system (RAS) (Remuzzi et al. 2006).

Recently, inhibitors of the sodium‐glucose transporter (SGLT)‐2 were found to elicit renoprotective effects in kidney disease in diabetic mice and rats (Gembardt et al. 2014; Ojima et al. 2015 Gallo et al. 2016;). Indeed, a large randomized controlled trial demonstrated a profound renoprotective effect of the SGLT2 inhibitor empagliflozin in addition to RAS blockade in patients with type 2 diabetes (Wanner et al. 2016). Empagliflozin somewhat improved glycemic control and hypertension, but these effects alone could not explain the immediate drop in the glomerular filtration rate (GFR) without any further decline during the following 3 years (Wanner et al. 2016). While the underlying mechanisms of renoprotection remain debated these findings raise the question whether SGLT2 inhibition can elicit renoprotective effects also in non‐diabetic kidney disease. To address this question, we tested empagliflozin versus vehicle treatment in a murine model of progressive non‐diabetic CKD based on oxalate‐induced nephropathy mimicking primary hyperoxaluria‐related nephrocalcinosis.

## Materials and Methods

### Animals and experimental design

Eight week‐old male C57BL/6N mice (Charles River Laboratories, Sulzfeld, Germany) were maintained under standard housing conditions with 10 mice per group, and free access to food and water. High‐oxalate diet was prepared by adding sodium oxalate (50 *μ*moles/g) to a calcium‐free diet or calcium‐free diet without sodium oxalate (control, both from Ssniff, Soest, Germany), as previously described (Knauf et al. 2013). Removal of calcium from the diet increases the amount of soluble oxalate available for absorption as previously shown (Knauf et al. 2013). Empagliflozin was dissolved in hydroxy ethyl cellulose (HEC) and administered by oral gavage (10 mg/kg, 200 *μ*L) to the oxalate‐treated mice once daily for 7 or 14 days, whereas the vehicle group was given the same volume of HEC alone. All animal experiments were performed in accordance with the European protection laws of animal welfare, and with approval by the local government authorities Regierung von Oberbayern (reference number: 55.2‐1‐54‐2532‐189‐2015).

### Measurement of plasma BUN, creatinine, and glucose

Mouse blood was collected from the tail vein of mice on the day of sacrifice. Blood samples were centrifuged at 8000 rpm for 8 min and plasma transferred to 1.5 mL plastic Eppendorf tubes, stored at −20°C until analysis. Plasma creatinine and blood urea nitrogen (BUN) were measured using the Creatinine FS kit and Urea FS kit from DiaSys Diagnostic Systems, Germany. Plasma glucose was measured using Glucose GOD FS kit (DiaSys) according to the protocol provided by the manufacturer.

### Measurement of urine oxalate, calcium, and glucose

Mouse urine samples were collected at different time points and stored at −20°C until analysis. Urine oxalate concentration was assessed with a colorimetric, enzymatic assay (Oxalate assay kit, Libios) in 96‐well plate according to the manufacturer's instructions. Urine calcium concentration was assessed using calcium colorimetric assay kit (Sigma‐Aldrich), and urinary glucose levels were determined using Glucose GOD FS kit (DiaSys).

### Assessment of renal histology

Kidneys were fixed in 4% formaldehyde solution for histology. 2 *μ*m thick sections were stained with periodic acid‐Schiff (PAS) reagent and for the kidney injury marker‐1 (KIM‐1). The renal tubular injury was evaluated by assessing the percentage of necrotic tubules, dilation tubules and presence of tubular casts (5× and 20× magnification). Fibrotic areas were identified by silver, *α*SMA and collagen 1 stain, and counted in 15 high power fields (hpf) per section using Image J software. Calcium oxalate (CaOx) crystals were visualized by Pizzolato staining and crystal deposit areas evaluated using Image J. All assessments were performed by an observer blinded to the experimental conditions.

### mRNA isolation and real‐time quantitative–PCR

Total RNA was isolated and purified from murine kidneys with a Qiagen RNA extraction kit (Qiagen, Germany) according to the manufacturer's instructions. mRNA was reverse transcribed to cDNA and carried out by reverse transcriptase (Superscript II, Invitrogen, USA). Real time RT‐PCR was performed using SYBR Green PCR master mix and analyzed with a Light Cycler 480 (Roche, Germany). The relative expression of target genes was normalized to 18s RNA control. The gene‐specific primer sequences were: KIM‐1 Forward, TCAGCTCGGGAATGCACAA; KIM‐1 Reverse, TGGTTGCCTTCCGTGTCTCT; TIMP‐2 Forward, CAGACGTAGTGATCAGAGCCAAA; TIMP‐2 Reverse, ACTCGATGTCTTTGTCAGGTCC; collagen 1*α*1 Forward, ACATGTTCAGCTTTGTGGACC; collagen 1*α*1 Reverse, TAGGCCATTGTGTATGCAGC; fibronectin‐1 Forward, GGAGTGGCACTGTCAACCTC; fibronectin‐1 Reverse, ACTGGATGGGGTGGGAAT; TNF*α* Forward, CCACCACGCTCTTCTGTCTAC; TNF*α* Reverse, AGGGTCTGGGCCATAGAACT; NLRP3 Forward, CCACAGTGTAACTTGCAGAAGC; NLRP3 Reverse, GGTGTGTGAAGTTCTGGTTGG; FSP‐1 Forward, CAG CAC TTC CTC TCT CTT GG; FSP‐1 Reverse, TTT GTG GAA GGT GGA CAC AA; TGF*β*2 Forward, GAT AAT TGC TGC CTT CGC CC; TGF*β*2 Reverse, GGC TGA GGA CTT TGG TGT GT; 18S RNA Forward, GCAATTATTCCCCATGAACG; 18S Reverse, AGGGCCTCACTAAACCATCC; IGFBP7 Forward, AAGAGGCGGAAGGGTAAAGC; IGFBP7 Reverse, TGGGGTAGGTGATGCCGTT. The data were analyzed using the 2^−ΔΔCT^ method.

### Transcutaneous measurement of glomerular filtration rate (GFR) in conscious mice

For GFR measurement, mice where anesthetized with isoflurane and a miniaturized imager device built from two light‐emitting diodes, a photodiode and a battery (Medibeacon, Mannheim, Germany) was mounted via a double‐sided adhesive tape onto the shaved animal's neck (Schreiber et al. 2012). The fluorescent dye FITC‐Sinistrin (150 mg/kg, Mannheim Pharma & Diagnostics GmbH, Germany) was injected intravenously, and each mouse was conscious and kept in a single cage throughout 1.5 h. After 1.5 h, the imager device was removed and the data analyzed using MPD Lab software (Mannheim Pharma & Diagnostics GmbH, Germany). The GFR (*μ*L/min) was calculated from the decrease of fluorescence intensity over time (i.e., plasma half‐life of FITC‐Sinistrin) using a two‐compartment model, body weight of mouse, and an empirical conversion factor (Schreiber et al. 2012).

### Statistical data analysis

Data are shown as means ± SEM between‐group differences were analyzed by two‐tailed Student *t*‐test or one‐way ANOVA, with Tukey's post hoc test, in which *P* < 0.05 was considered statistically significant.

## Results

### Empagliflozin has no effect on renal oxalate crystal deposition

**Figure 1:**
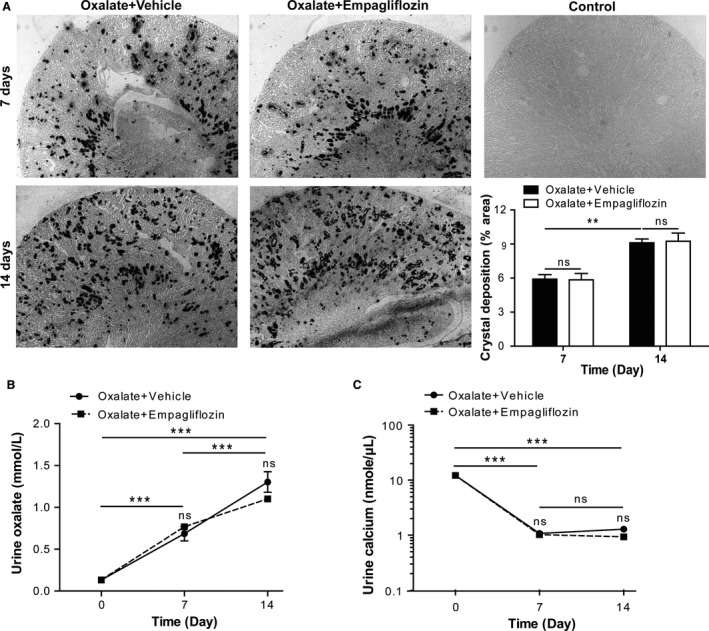
Empagliflozin treatment has no effect on oxalate crystal deposition in C57BL/6N mice. C57BL/6N male mice were fed a high‐oxalate diet in combination with empagliflozin or vehicle for 7 and 14 days. (A) Kidney sections were quantified for CaOx crystal deposits using a light microscope (5× magnification), (B) urine oxalate and (C) urine calcium excretion were measured at indicated time points. Data are mean ± SEM from 10 mice in each group. *P < 0.05, **P < 0.01 and ***P < 0.001 versus control groups. Ns, not significant.

Feeding a diet rich in oxalate but depleted of calcium to C57BL/6N mice represents a reliable model of non‐diabetic nephrocalcinosis and progressive CKD (Knauf et al. 2013; Mulay 2016). Indeed, oxalate feeding resulted in the progressive deposition of calcium oxalate (CaOx) crystals in the renal medulla and cortex after 7 and 14 days (Fig. 1A). Treatment with empagliflozin had no effect on CaOx crystal deposition compared to vehicle, as indicated by Pizzolato staining and the % area of crystal deposition (Fig. 1A). Urinary oxalate levels significantly increased over time following oxalate feeding (Fig. 1B). Urinary calcium levels rather declined upon feeding a calcium‐depleted diet (Fig. 1C), but empagliflozin treatment did neither affect oxaluria nor calciuria (Fig. 1B and 1C). This enabled us to assess the impact of empagliflozin treatment in comparison to untreated control mice with identical oxalate‐induced kidney injury.

### Empagliflozin does not attenuate the progressive loss of renal function

**Figure 2:**
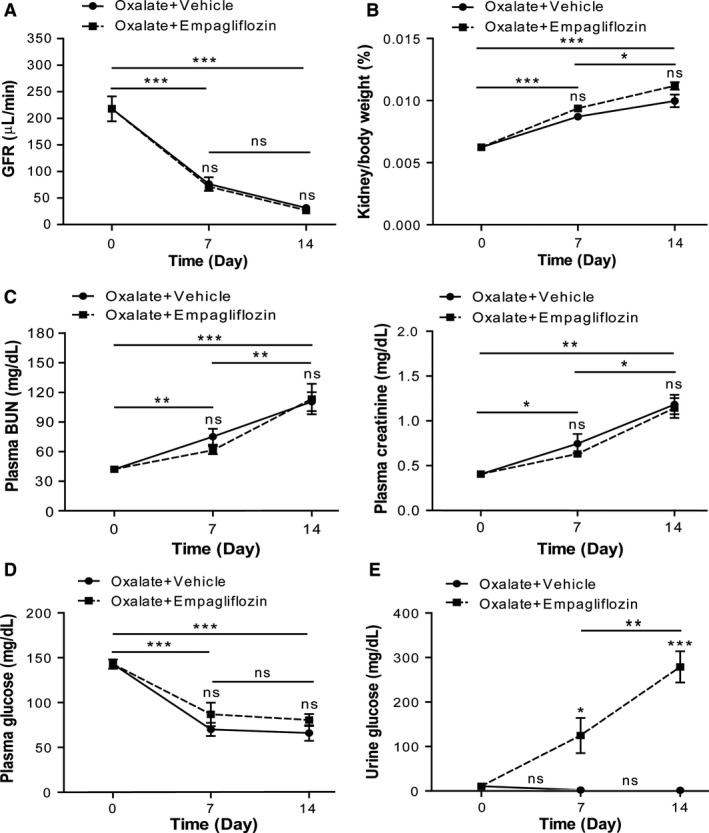
Empagliflozin does not prevent from oxalate‐induced renal dysfunction. C57BL/6N male mice received high‐oxalate diet with empagliflozin or vehicle daily for 7 and 14 days. (A) Glomerular filtration rate (GFR), (B) kidney/body weight ratio, (C) plasma BUN and plasma creatinine levels, and (D) plasma and (E) urine glucose levels were measured at indicated time points. Data are mean ± SEM from 10 mice in each group. *P < 0.05, **P < 0.01 and ***P < 0.001 versus control groups. Ns, not significant.

Hyperoxaluria‐induced nephrocalcinosis resulted in a progressive decline in the GFR (Fig. 2A), and in a significant increase in plasma BUN and creatinine levels (Fig. 2C) after 7 and 14 days. Treatment with the SGLT‐2 inhibitor empagliflozin did not attenuate the progressive loss of renal function, as indicated by GFR, plasma BUN, and creatinine compared to vehicle treatment (Fig. 2A and C). No difference in the kidney/body weight ratio between empagliflozin and vehicle treatment was observed in oxalate‐treated mice (Fig. 2B). However, empagliflozin had no significant effect on serum glucose levels (Fig. 2D), despite inducing significant glucosuria compared to vehicle‐treated control mice (Fig. 2E). Together, these data imply that inhibiting SGLT‐2 with empagliflozin does not affect the progressive decline of renal function in CaOx crystal‐induced nephropathy.

### Empagliflozin and renal pathology in CaOx crystal‐induced CKD

**Figure 3:**
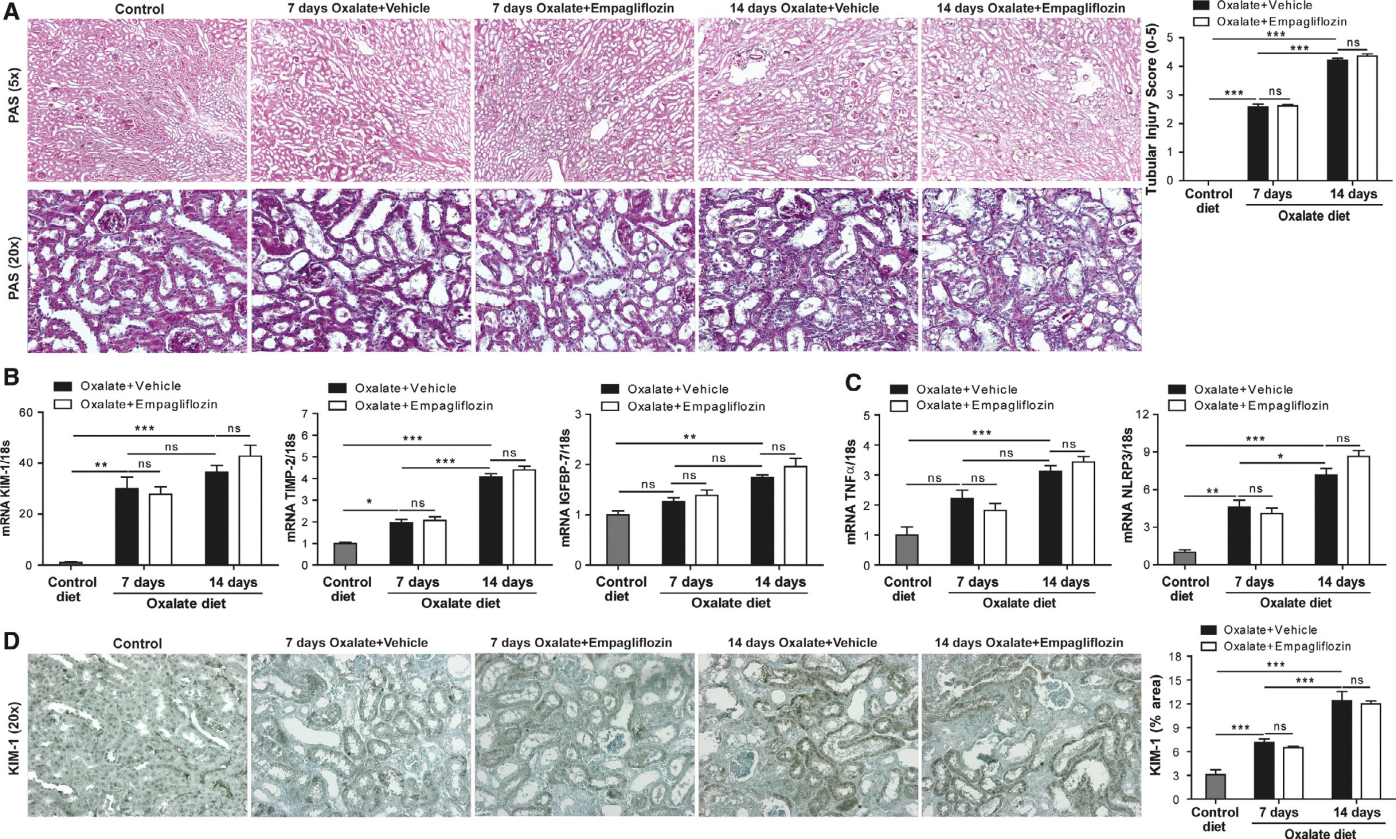
Empagliflozin has no effect on oxalate nephropathy injury in C57BL/6N mice. C57BL/6N male mice received high‐oxalate diet plus vehicle or high‐oxalate diet with empagliflozin for 7 or 14 days, respectively. (A) PAS staining at various time points and quantification (5× and 20× magnification). Gene expression of the renal injury markers kidney injury molecule 1 (KIM‐1), metallopeptidase inhibitor 2 (TIMP‐2), and insulin like growth factor binding protein 7 (IGFBP7) (B), as well as TNF *α* and NLRP3 (C) was analyzed using RT‐qPCR at different time points. (D) KIM‐1 immunostaining of kidney sections on day 7 and 14 (20× magnification). Data are mean ± SEM from 10 mice in each group. *P < 0.05, **P < 0.01 and ***P < 0.001 versus control groups. Ns, not significant.

Tubular atrophy and interstitial fibrosis are hallmark features of progressive CKD regardless of the underlying disease trigger. We next examined the effect of empagliflozin on renal injury in CaOx crystal‐induced CKD at various time points. Renal CaOx crystal deposition was associated with progressive tubular atrophy in vehicle‐ and empagliflozin‐treated mice after 7 and 14 days compared to control diet, as indicated by PAS staining and tubular injury score (Fig. 3A). Intrarenal mRNA expression of the kidney injury marker KIM‐1, metallopeptidase inhibitor‐2 (TIMP‐2), and insulin like growth factor binding protein 7 (IGFBP7) (Fig. 3B), as well as that of the pro‐inflammatory mediators tumor necrosis factor (TNF)*α* and the inflammasome protein NLRP3 (Fig. 3C) was increased in oxalate‐fed mice compared to control diet. This was also the case for the protein expression of KIM‐1 in hyperoxaluric mice compared to control diet (Fig. 3D). However, empagliflozin had no renoprotective effect regarding tubular injury and inflammation in CaOx crystal‐induced nephropathy.

**Figure 4:**
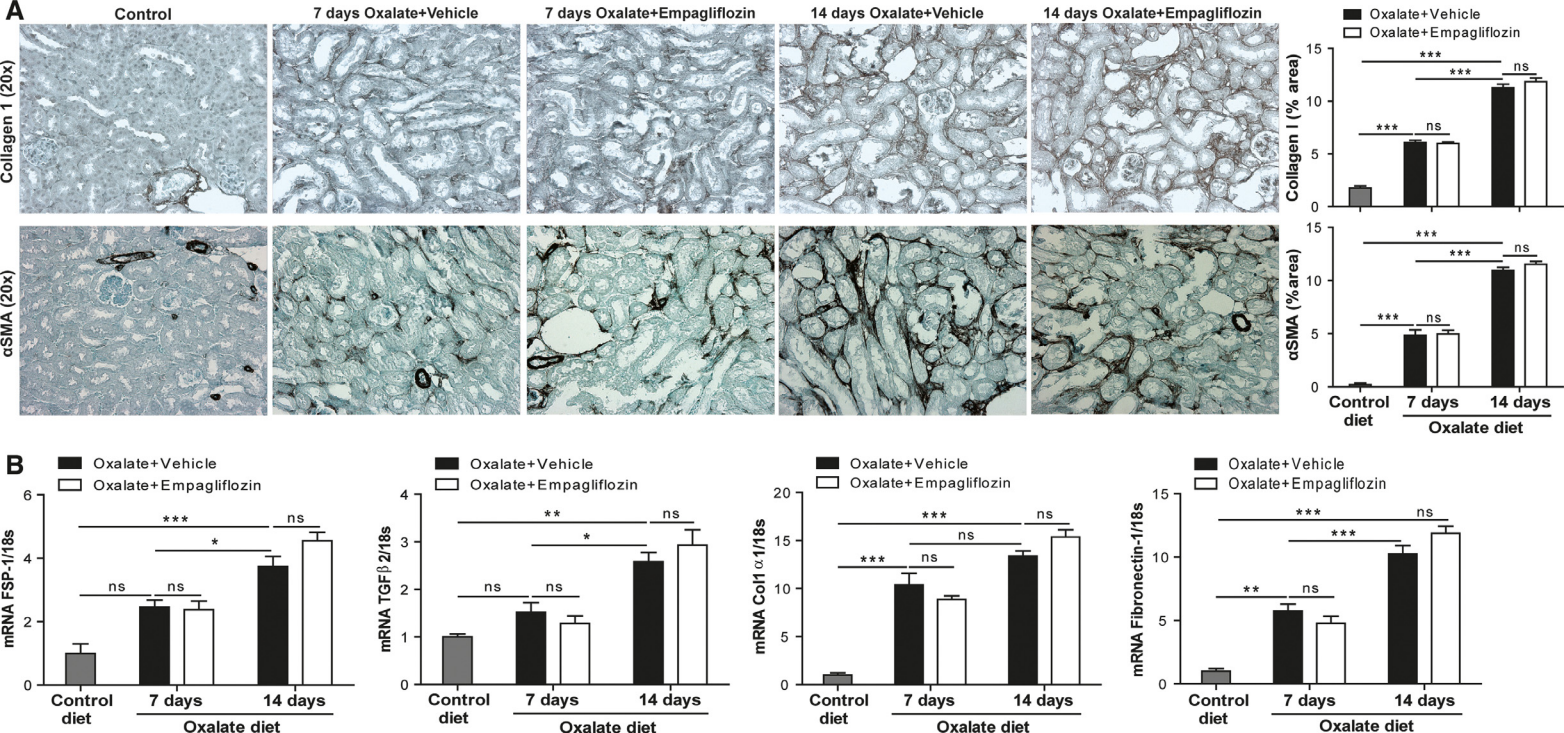
Fibrosis of CaOx‐induced CKD with or without empagliflozin in C57BL/6N mice. C57BL/6N male mice received high‐oxalate diet plus vehicle or high‐oxalate diet with empagliflozin for 7 or 14 days. (A) Collagen 1*α*1 and *α* SMA staining at various time points, and quantification (20× magnification). (B) Gene expression of the kidney fibrosis markers fibroblast‐specific protein (FSP)‐1, transforming growth factor (TGF)‐*β*2, collagen 1*α*1 (Col1*α*1), and fibronectin‐1 was analyzed using RT‐qPCR at different time points. Data are mean ± SEM from 10 mice in each group. *P < 0.05, **P < 0.01 and ***P < 0.001 versus control groups. Ns, not significant.

Finally, progressive interstitial fibrosis was assessed by *α*SMA and collagen 1 staining (Fig. 4A), silver staining (Fig. S1), and mRNA expression profiling for the fibrosis marker fibroblast‐specific protein (FSP)‐1, transforming growth factor (TGF)*β*2, collagen 1*α*1 (Col1*α*1), and fibronectin‐1 (Fig. 4B). When compared with vehicle‐treated mice, empagliflozin did not alter the progression of fibrosis in CaOx crystal‐related nephrocalcinosis. Thus, SGLT2 inhibition with empagliflozin does not affect progressive CKD in CaOx crystal‐induced nephropathy.

## Discussion

We speculated that SGLT2 inhibition with empagliflozin might have some renoprotective effects in oxalate‐induced nephrocalcinosis. However, our data disprove this concept and suggest that the renoprotective effects of SGLT2 inhibition seen in diabetic kidney disease, does not occur in this non‐diabetic form of progressive CKD driven by primary tubulointerstitial injury.

The renoprotective effects of the SGLT2 inhibitor empagliflozin in kidney disease of type 2 diabetic patients are impressive, and even exceed those reported for RAS inhibitors (Remuzzi et al. 2006; Wanner et al. 2016). But the potential underlying mechanisms‐of‐actions are numerous and still a subject of debate (Vallon 2015). At first, SGLT2 inhibitors are anti‐diabetic drugs. Empagliflozin reduced Hba1c in major clinical trials on average by 0.62% (Wanner et al. 2016). Glycemic control with other drugs for up to 3, 7 years in the Action to Control Cardiovascular Risk in Diabetes (ACCORD) trial resulted in decreased albuminuria, but not in progressive CKD after 5 years (Ismail‐Beigi et al. 2010). Controlling glycemic patients had no effect on the pre‐specified renal outcomes in the UK Prospective Diabetes Study (UKPDS) trial (Bilous 2008). These data imply that empagliflozin's anti‐diabetic effect alone does not account for its profound renoprotective effects. Similarly, empagliflozin reduced systolic and diastolic blood in major clinical trials on average by 4.19 and 1.88 mmHg, respectively (Liakos et al. 2014). However, an even more rigorous blood pressure control in patients with diabetes did not reveal renoprotective effects comparable to those seen in the EMPA‐REG trial (UK Prospective Diabetes Study Group, 1998; de Galan et al. 2009; Group, A.S., et al. 2010; Ismail‐Beigi et al. 2012). Reactivation of the tubuloglomerular feedback (TGF) is another possible mechanism‐of‐action, because glomerular hyperfiltration and hypertension in diabetes are rather a direct consequence of SGLT2‐mediated increased sodium reabsorption in the proximal tubule, which reduces sodium delivery in the *macula densa*, a process that deactivates the TGF and causes vasodilation of the afferent glomerular arteriole (Carlstrom et al. 2015; Skrtic and Cherney 2015; Vallon 2015). This mechanism seems specific for hyperglycemic states, because only increased glucose filtration would serve as a trigger and driver of decreased sodium delivery to the *macula densa* (Anders et al. 2016). In contrast, in the absence of hyperglycemia SGLT2 is not induced and contributes only minimally to proximal sodium reabsorption, hence, SGLT2 inhibition may not significantly increase distal sodium delivery and elicit hemodynamic effects on glomerular filtration.

In contrast to the critical role of SGLT2 in the pathogenesis of progressive diabetic kidney disease, our data imply that the progression of oxalate nephropathy does not involve SGLT2. Chronic oxalosis is driven by intrarenal supersaturation of oxalate and calcium ions that promote crystal formation inside the tubular lumen, especially in segments of calcium secretion and urine concentration (Cochat and Rumsby 2013). Calcium oxalate crystal adhesion depends on the luminal expression of several crystal adhesion molecules, such as CD44, annexin II, and osteopontin (Asselman et al. 2003) which are induced by TNF receptor signaling in tubular epithelial cells (Mulay 2016). Crystal adhesion promotes further crystal growth to crystal plugs that obstruct the tubular lumen leading to intrarenal nephron obstruction (Mulay 2016). Tubule obstruction is then followed by tubular atrophy and replacement with fibrous tissue. Hence, progressive nephrocalcinosis is associated with a progressive decline in renal function, as illustrated by a decline in GFR (Mulay 2016). SGLT2 does not seem to interfere with any of these known pathomechanisms, probably mainly because of the TGF deactivation, and the consecutive glomerular hypertension and hyperfiltration that are not features of this form of progressive CKD. However, glomerular hypertension and hyperfiltration may not respond to SGLT2 inhibition in the absence of diabetes. For example, Zhang et al. (2016) evaluated the effects of the SGLT2 inhibitor dapagliflozin in a rat model of renal mass ablation by 5/6 nephrectomy, which induces progressive glomerulosclerosis. In the absence of hyperglycemia dapagliflozin treatment did not show any renoprotective effect (Zhang et al. 2016).

In summary, SGLT2 inhibition with empagliflozin does not affect CKD progression in oxalate‐related nephrocalcinosis. These data do not support SGLT2 as a therapeutic target in non‐diabetic forms of CKD, especially when driven by tubulointerstitial injury.

## Conflict of Interest

The authors have nothing to disclose.

## Data Accessibility

## Supporting information




**Fig S1.** Fibrosis in oxalate nephropathy with or without empagliflozin.
