# Supplementary figures and images for: Sodium glucose transporter‐2 inhibition has no renoprotective effects on non‐diabetic chronic kidney disease
Source: Physiol Rep. 2017 Mar 31;5(7):e13228. doi: 10.14814/phy2.13228 (PMC5392518; doi:10.14814/phy2.13228)

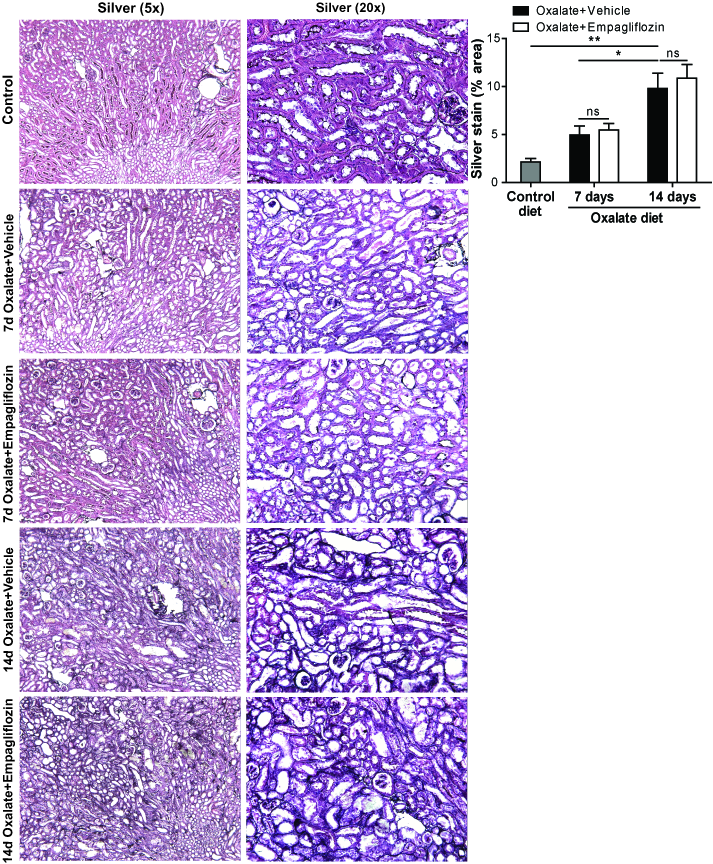

Supplement: Supplementary file 1 — Fig S1. Fibrosis in oxalate nephropathy with or without empagliflozin. [file PHY2-5-e13228-s001.tif]
