# Supplementary material for: Sodium glucose transporter‐2 inhibition has no renoprotective effects on non‐diabetic chronic kidney disease
Source: Physiol Rep. 2017 Mar 31;5(7):e13228. doi: 10.14814/phy2.13228 (PMC5392518; doi:10.14814/phy2.13228)
Supplement: Supplementary file 2 [file PHY2-5-e13228-s002.docx]

**Supplementary figure 1. Fibrosis in oxalate nephropathy with or without empagliflozin.**

C57BL/6N male mice received high oxalate diet plus vehicle or high oxalate diet with empagliflozin for 7 or 14 days. Silver staining at various time points, and quantification (5x and 20x magnification). Ns = not significant.
